# Effects of HMG on Superovulation Efficacy in Kazakh Sheep and Screening of Plasma Reproductive Hormones and Metabolic Markers

**DOI:** 10.3390/ani16152326

**Published:** 2026-07-30

**Authors:** Peng Zhang, Nana Yang, Baoyue Cui, Meng Li, Xinglong Wu, Xiangyun Li

**Affiliations:** College of Animal Science and Technology, Hebei Technology Innovation Center of Cattle and Sheep Embryo, Hebei Agricultural University, Baoding 071000, China; zp163163123123@163.com (P.Z.); 17693378189@163.com (N.Y.); 17853978982@163.com (B.C.); limengtudou@163.com (M.L.); wuxl32@hebau.edu.cn (X.W.)

**Keywords:** superovulation, HMG, metabolomics, FSH

## Abstract

Superovulation and embryo transfer techniques are effective strategies for improving reproductive efficiency in sheep flocks. To characterize changes in plasma metabolites and reproductive hormones following HMG or FSH superovulatory treatment, and to screen for biomarkers closely associated with embryo quality, we employed liquid chromatography-based untargeted metabolomics and enzyme-linked immunosorbent assay (ELISA) to analyze plasma samples from donor ewes on day 6 after hormone administration. This study aimed to identify small-molecule biomarkers affecting embryo quality under different hormonal regimens. A total of 46 Kazakh ewes were allocated into two groups: the HMG group received HMG treatment, while the FSH group received FSH treatment; both groups were superovulated using a tapering dose regimen administered via intramuscular injection. Our results show that the average number of viable embryos in the HMG group (5.36 ± 3.29, *n* = 11) was significantly higher than that in the FSH group (2.91 ± 2.60, *n* = 35) (*p* < 0.05). Plasma progesterone concentrations differed significantly between the two groups (*p* = 0.01). Metabolomic analysis identified three key differential metabolites: 5-fluorouridine, ADP, and hexadecanoic acid. Functional enrichment analysis revealed 45 significantly enriched metabolic pathways. In conclusion, HMG effectively improves sheep embryo quality by modulating the levels of small-molecule metabolites associated with embryonic development.

## Simple Summary

To investigate the effects of superovulation treatment with human menopausal gonadotropin on increasing the number of available embryos and optimizing embryo morphological quality in sheep, and to screen for biomarkers closely associated with embryo quality, thereby providing an efficient and stable novel theoretical basis and technical support for improving sheep reproductive efficiency. Metabolic analysis showed that human menopausal gonadotropin treatment induced a significant upregulation of ADP, a metabolite that maintains embryonic energy homeostasis, and significantly downregulated pentfluorouridine and hexadecanoic acid, metabolites associated with embryonic death. These findings indicate that human menopausal gonadotropin improves embryonic energy supply and alleviates developmental toxicity and lipotoxic injury by upregulating ADP and downregulating pentfluorouridine and hexadecanoic acid, thereby enhancing embryo quality. This ultimately improves reproductive efficiency and provides economic benefits for the livestock industry.

## 1. Introduction

Sheep serve as an irreplaceable model species for reproductive biotechnology research in ruminants and play a pivotal role in the animal husbandry industry. Historically, most key technologies associated with ruminant embryo engineering—such as multiple ovulation and embryo transfer (MOET)—were first developed and optimized in sheep, and were subsequently extended to other livestock species, including cattle and goats [1]. As the core component of the MOET system, superovulation (SOV) stimulates the ovaries of ewes via exogenous gonadotropins, inducing synchronous development and ovulation of multiple follicles. This approach allows for the collection of high-quality oocytes and embryos far exceeding natural ovulation numbers (typically more than fivefold) within a single estrous cycle, thereby markedly enhancing the reproductive efficiency of elite ewes and accelerating genetic improvement [2,3,4,5].

Since the 1930s, various gonadotropins have been employed to regulate ovulation rate in sheep, including follicle-stimulating hormone (FSH), equine chorionic gonadotropin (eCG), human chorionic gonadotropin (hCG), gonadotropin-releasing hormone (GnRH), and human menopausal gonadotropin (HMG). Among these, eCG and porcine/ovine FSH (p/oFSH) are the most commonly used hormonal preparations in current sheep superovulation protocols. As a glycoprotein hormone secreted by the pituitary gland under the regulation of hypothalamic GnRH, FSH plays a dominant role in follicular development and oocyte selection. In conventional superovulation regimens, FSH is typically administered via intramuscular injection in a decreasing dose regimen over 6–8 injections (at 12 h intervals) during days 3–5 of the follicular phase, yielding an average of approximately 15.9 ovulations, 10.7 recovered embryos, and 5.5 transferable embryos [6,7,8].

However, FSH has notable limitations. Its short half-life (approximately 3–4 h) necessitates frequent administration, making the protocol cumbersome. Moreover, individual superovulatory responses are highly variable and susceptible to environmental and physiological status. FSH-based protocols are often associated with insufficient luteal function and poor embryo quality, leading to a low rate of transferable embryos and potentially impairing the long-term reproductive performance of donor ewes [9,10,11,12]. Although some studies have explored simplified regimens using single-injection FSH or FSH combined with eCG, these approaches have shown no significant advantages in ovulation number, embryo recovery rate, or fertilization rate compared with the traditional multiple-injection FSH protocol (*p* > 0.05) [10,11], indicating that the inherent biological limitations of FSH are difficult to overcome simply by altering administration frequency.

In contrast, human menopausal gonadotropin (HMG)—a complex hormonal preparation extracted from the urine of postmenopausal women—exhibits both FSH and LH biological activities. Since its first successful application in bovine superovulation in 1982 [13], HMG has demonstrated greater application potential than FSH. Studies have shown that HMG not only yields ovulation numbers and transferable embryo counts comparable to, or even higher than, those of FSH [14,15], but also significantly improves embryo quality [16,17]. Its half-life is approximately 18 h [18], which supports more simplified administration regimens. For example, a single injection of 450–600 IU HMG can effectively induce superovulation in Japanese Black cattle [17], and a total dose of 900 IU administered twice daily for four consecutive days significantly increases the proportion of high-quality embryos [16]. These advantages position HMG as a strong candidate to replace FSH in optimizing superovulation protocols.

Based on our preliminary experimental observations—although there were no significant differences in the total number of recovered oocytes/embryos between the FSH and HMG treatment groups—the HMG group exhibited a higher proportion of transferable embryos with better morphological quality. We therefore hypothesized that HMG may regulate the dynamics of reproductive hormones and the metabolic microenvironment in ewes, thereby influencing oocyte maturation and early embryonic development. Furthermore, few studies have investigated the mechanisms by which HMG increases the number of viable embryos during superovulation in Kazakh sheep. Thus, the present study aimed to systematically compare the effects of FSH and HMG superovulation protocols on plasma reproductive hormone profiles and metabolomic characteristics in sheep following artificial insemination. Our objectives were to clarify the regulatory mechanisms by which different hormonal treatments affect maternal reproductive endocrine and metabolic homeostasis, and to screen for metabolic biomarkers closely associated with embryo quality. This study will provide a theoretical basis and technical support for establishing an efficient, stable, and low-stress superovulation strategy in sheep.

## 2. Materials and Methods

### 2.1. Experimental Site and Animals

The present study was conducted at Zhaosu Horse Farm in Zhaosu County, Ili Kazakh Autonomous Prefecture, Xinjiang, China. A total of 46 Kazakh ewes, aged 2–4 years and weighing 35–45 kg, were selected. All ewes were in good health, exhibited normal estrous cycles, and were free from reproductive tract diseases. The ewes were housed indoors. Their diet consisted of natural forage and corn silage as roughage, supplemented with a complete formula concentrate. Roughage and concentrate were mixed before feeding, and water and mineral salt blocks were available ad libitum. Animals were fed twice daily at fixed times. All experimental procedures were approved by the Animal Care and Use Committee of Hebei Agricultural University (Approval No. 2024-027).

### 2.2. Estrus Synchronization and Superovulation

Before the start of the trial, following confirmation of normal estrous cyclicity in the ewes, estrous synchronization was performed on all animals (*n* = 46). A normal estrous cycle in ewes was defined as an inter-estrus interval of 16–19 days, with a stable interval across three consecutive cycles. Estrus was detected at each cycle using teaser rams. The teaser rams were fitted with aprons to prevent copulation while allowing full expression of sexual behavior. When a ewe entered estrus, alterations in her behavioral, physiological, and chemical signals attracted the attention of the teaser ram. Ewes in estrus exhibited behaviors such as repeatedly approaching the ram and standing immobile to be mounted, while the teaser ram responded with sniffing, chasing, and mounting attempts. The observation of standing estrus, characterized by the ewe accepting mounting by the ram, was taken as confirmation of normal estrus. Only ewes that displayed estrus and accepted mounting by the teaser ram in each of three consecutive cycles were considered to have normal estrous cycles. Subsequently, all ewes underwent synchronized estrus treatment by intravaginal insertion of progesterone-releasing sponges containing 45 mg of fluorogestone acetate (Shanghai Muqi Animal Husbandry Technology Co., Ltd., Shanghai, China). The day of sponge insertion was designated as D0.

For the FSH treatment group (*n* = 35), decreasing doses of FSH (porcine origin, 500 IU/vial; Ningbo Sansheng Biological Technology Co., Ltd., Ningbo, China) were administered intramuscularly twice daily (9:00 AM and 9:00 PM) from D10 to D13, totaling six injections with a cumulative dose of 420 IU (90, 90, 70, 70, 50, 50 IU). At the time of the fifth FSH injection, 1 mL of prostaglandin (PG, 2 mL: 0.2 mg/vial) was co-administered to regulate follicular development and promote ovulation, acting as a key mediator to increase preovulatory follicle numbers, coordinate estrus, and prepare for fertilization and implantation. The sponges were removed at 9:00 AM on D13. Estrus was detected at 9:00 AM on D14, and artificial insemination was performed at 8:00 PM on the same day. Feed was withheld on D17, and both feed and water were withheld on D18. On the morning of D19, embryos were recovered from the uterus via a surgical approach.

**Figure 1:**
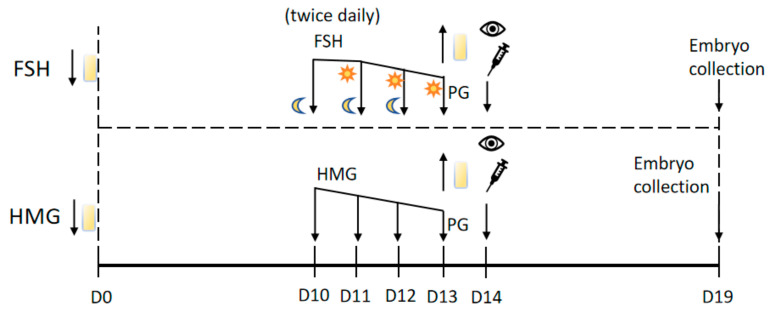
Schematic diagram of the superovulation protocol for Kazakh ewes. This schematic illustrates the specific treatment regimens for the FSH and HMG groups.

For the HMG treatment group (*n* = 11), decreasing doses of HMG (75 IU/vial; Ningbo Renjian Pharmaceutical Co., Ltd., Ningbo, China) were administered intramuscularly once daily at 9:00 AM from D10 to D13, with a total dose of 525 IU (225, 150, 75, 75 IU). Sponges were removed at 9:00 AM on D13, and 1 mL of PG was injected at 8:00 PM on the same day. Estrus was detected at 9:00 AM on D14, and laparoscopic artificial insemination was performed at 8:00 PM. Feed was withheld on D17, and both feed and water were withheld on D18. On the morning of D19, embryos were recovered from the uterus via a surgical approach (Figure 1).

Estrus was assessed in all ewes at 24 h after sponge removal. Ewes that exhibited estrus underwent artificial insemination, whereas those that did not exhibit estrus received an intramuscular injection of 50 IU FSH or 75 IU HMG, according to their group allocation. For sponge insertion, the sponges were treated with penicillin sodium (1,600,000 IU/vial; Hebei Yuanzheng Pharmaceutical Co., Ltd., Shijiazhuang, China), and the tail strings were trimmed to a suitable length. The external vulva was cleansed with a penicillin-saline solution. The sponge was manually rolled, inserted into the vagina, passed through the vaginal isthmus, and the trimmed string was left protruding from the vulva. For removal, the protruding string was grasped and pulled gently but firmly to withdraw the sponge, followed by vaginal lavage.

### 2.3. Fixed-Time Insemination

Fresh semen was collected from six healthy Kazakh rams and immediately transferred into sterile test tubes maintained in a water bath at 30 °C. A drop of semen was placed onto a preheated glass slide using a dropper. Sperm motility and concentration were assessed using a hemocytometer under a microscope equipped with a heating stage (Shanghai Muqi Animal Husbandry Technology Co., Ltd., Shanghai, China). Normal fresh semen exhibited characteristic cloud-like motility. The total sperm number per ejaculate ranged from 3.5 to 6.0 × 10^9^. Only ejaculates with sperm motility ≥ 70% were used for subsequent procedures. The selected semen was diluted sixfold with pre-warmed skimmed milk at 30 °C to adjust the final sperm concentration to 1 × 10^8^ sperm per insemination dose. Artificial insemination was then performed using the freshly diluted semen.

All ewes were fasted for 24–36 h and deprived of water for 12–18 h before insemination to reduce rumen volume and facilitate laparoscopic visualization (Tonglu Yushi Medical Device Co., Ltd., Hangzhou, China). Artificial insemination was performed at 34–40 h after vaginal sponge removal. Ewes were anesthetized with Xylazine Hydrochloride Injection (2 mL: 0.2 g per vial; Dunhua Shengda Veterinary Pharmaceutical Co., Ltd., Dunhua, China). The stock solution was diluted 20-fold with sterile saline, and 0.3 mL of the diluted solution was administered intravenously.

During insemination, ewes were positioned in dorsal recumbency on the operating table with the abdomen tilted upward. The hair around the mammary gland and along the bilateral abdominal midline was shaved, and the surgical area was disinfected with tincture of iodine (available iodine 5.0 g/L ± 0.5 g/L; Shandong Likang Disinfection Technology Co., Ltd., Dezhou, China) and 75% medical alcohol. Peritoneal puncture was performed at 3–4 cm lateral to the abdominal midline using a trocar, followed by laparoscope insertion. The uterine horn was gently exteriorized for approximately 3 cm using uterine forceps. A penicillin-streptomycin solution was sprayed onto the uterine horn to maintain moisture and provide local anti-inflammatory protection. Finally, 0.3 mL of diluted semen was injected into each uterine horn using a 1 mL syringe.

### 2.4. Embryo Recovery and Quality Grading Criteria

On Day 19 at 09:00 h, surgical uterine flushing was performed for embryo recovery. The donor ewe was placed in dorsal recumbency on a surgical table. Anesthesia was induced by intravenous injection of Lumianning^®^ (xylazine hydrochloride injection, 2 mL: 0.2 g per vial), which was diluted 10-fold with sterile normal saline and administered into the jugular vein at a dose of 0.1 mL per 25 kg body weight. The surgical site was clipped, disinfected with iodophor, and subsequently wiped with 75% ethanol to remove residual iodine. A 4 cm vertical skin incision was made along the ventral midline, approximately 8 cm caudal to the base of the udder, at a relatively avascular site. The underlying muscle, adipose tissue, and peritoneum were bluntly dissected to enter the abdominal cavity. The uterus was exteriorized by gently grasping it with the index and middle fingers, and saline-moistened sterile gauze was packed around the uterus to stabilize it. Prior to flushing, a non-crushing intestinal clamp was applied across the uterine bifurcation to isolate one horn. A puncture hole approximately 1.5 mm in diameter was created at the base of the uterine horn using the tip of a small hemostatic forceps. An embryo recovery catheter was inserted into the uterine lumen through this opening. At the thinner, distal portion of the same horn, an indwelling catheter was inserted via direct puncture to serve as the infusion port for the flushing medium. Each uterine horn was flushed with 30 mL of pre-warmed flushing medium. After flushing, the uterus was carefully returned to the abdominal cavity, and the incision was closed in layers. The muscle layer was closed with a simple continuous suture pattern, and the dermal layer was apposed with interrupted sutures. Finally, erythromycin ointment was applied topically to the wound for its anti-inflammatory and antimicrobial effects.

Embryo quality is classified into four morphological grades (A, B, C, and D) based on standard criteria.

Grade A: Embryos exhibit intact morphology with a clearly defined, spherical outline. Blastomeres are uniform in size, densely arranged, and display moderate tone and transparency. No adherent cells or fluid-filled vesicles are present. Grade B: Embryos present a distinct outline with satisfactory cell density and coloration. A small number of adherent cells or vesicles may be observed, and the proportion of degenerated cells ranges from 10% to 30%. Grade C: Embryos show a blurred outline, darkened appearance, and loosely organized structure. A notable number of free cells or vesicles is visible, with degenerated cells accounting for 30% to 50%. Grade D: This category includes fertilized oocytes at the 16-cell stage or earlier, as well as embryos exhibiting fragmentation, poorly defined outlines, disorganized structure, or a degenerated cell proportion exceeding 50%. Grade A and B embryos are suitable for fresh transfer and cryopreservation. Grade C embryos may only be used for fresh transfer and are not eligible for cryopreservation. Grade D embryos are considered non-viable. In the present study, only Grade A and B embryos were selected for transfer.

### 2.5. Blood Sample Collection

On day 6 after artificial insemination (at 9:00 AM), blood samples were collected from the Kazakh ewes via jugular venipuncture. Approximately 5 mL of blood was drawn into heparinized vacuum tubes using disposable blood collection devices (Shandong Aosaite Medical Devices Co., Ltd., Heze, China). All blood samples were centrifuged at 3000 rpm for 15 min at 4 °C using a refrigerated centrifuge (Thermo Fisher Scientific (China) Co., Ltd., Shanghai, China). The resulting plasma supernatant was transferred into sterile, nuclease-free 1.5 mL microcentrifuge tubes (Axygen, Union, CA, USA) using pipettes (Eppendorf, Hamburg, Germany), and the tubes were clearly labeled. All plasma samples were stored at −80 °C to maintain sample integrity until further metabolomic analysis.

### 2.6. Determination of Hormone Concentrations

Concentrations of reproductive hormones, including progesterone (PROG), estradiol (E2), prostaglandin F2α (PGF2α), FSH, and LH, were determined using commercial ELISA kits (Shanghai Jianglai Biotechnology Co., Ltd., Shanghai, China) according to the manufacturer’s instructions.

### 2.7. Metabolite Extraction

Aliquots of plasma (100 μL) were transferred into microcentrifuge tubes and resuspended in prechilled 80% methanol by vigorous vortexing. The samples were incubated on ice for 5 min and then centrifuged at 15,000× *g* for 20 min at 4 °C. An aliquot of the supernatant was diluted with LC-MS grade water to a final methanol concentration of 53%. The diluted samples were then transferred to fresh tubes and centrifuged again at 15,000× *g* for 20 min at 4 °C. Finally, the supernatant was collected for LC-MS/MS analysis.

### 2.8. LC-MS/Ms Analysis

UHPLC-MS/MS analysis was performed using a Vanquish UHPLC system (Thermo Fisher, Bremen, Germany) coupled to an Orbitrap Q Exactive™ HF or Q Exactive™ HF-X mass spectrometer (Thermo Fisher, Germany). Chromatographic separation was achieved on a Hypersil Gold column (100 × 2.1 mm, 1.9 μm) at a flow rate of 0.2 mL/min using a 12 min linear gradient. The mobile phases consisted of Eluent A (0.1% formic acid in water) and Eluent B (methanol). The gradient program was as follows: 2% B for 1.5 min, 2–85% B over 3 min, 85–100% B over 10 min, 100–2% B at 10.1 min, and held at 2% B until 12 min. The mass spectrometer was operated in both positive and negative ion modes with the following parameters: spray voltage, 3.5 kV; capillary temperature, 320 °C; sheath gas flow rate, 35 psi; auxiliary gas flow rate, 10 L/min; S-lens RF level, 60; and auxiliary gas heater temperature, 350 °C.

### 2.9. Data Analysis

Metabolite annotation was performed using the KEGG (https://www.genome.jp/kegg/pathway.html (accessed on 25 June 2025)), HMDB (https://hmdb.ca/metabolites (accessed on 25 June 2025)), and LIPIDMaps (http://www.lipidmaps.org/ (accessed on 25 June 2025)) databases. Multivariate statistical analyses, including principal component analysis (PCA) and partial least squares discriminant analysis (PLS-DA), were conducted using the metaX software platform version 2.0.0. Univariate analysis (Student’s *t*-test) was applied to calculate *p*-values. Differential metabolites were selected based on the following criteria: variable importance in projection (VIP) > 1, *p* < 0.05, and fold change ≥ 2 or ≤0.5. Volcano plots were generated using the ggplot2 package in R to visualize metabolites of interest based on log2(fold change) and −log10(*p*-value). Heatmaps were constructed using the Pheatmap package after z-score normalization of differential metabolite intensities. Pearson correlation analysis between differential metabolites was performed using the cor() function in R, with statistical significance assessed by cor.mtest() (*p* < 0.05), and correlation plots were generated using the corrplot package. Functional annotation and pathway enrichment analysis were performed using the KEGG database; pathways were considered enriched when the ratio x/n > y/N, and statistically significant enrichment was declared when *p* < 0.05.

Pregnancy rate was calculated as Number of pregnant ewes/Number of inseminated ewes. Lambing rate was calculated as Number of lambing ewes/Number of pregnant ewes.

## 3. Results

### 3.1. Analysis of Results Regarding Superovulation and Embryo Count

The number of embryos recovered was recorded on the day of uterine flushing. A total of 59 viable embryos were collected from the 11 Kazakh ewes in the HMG group, averaging 5.36 ± 3.29 viable embryos per ewe. In comparison, 102 viable embryos were collected from the 35 Kazakh ewes in the FSH group, averaging 2.91 ± 2.60 viable embryos per ewe (*p* < 0.05). Detailed embryo recovery data are presented in Table 1.

### 3.2. Non-Targeted Metabolomics Analysis of Kazakh Sheep Plasma During Superovulation

**Figure 2:**
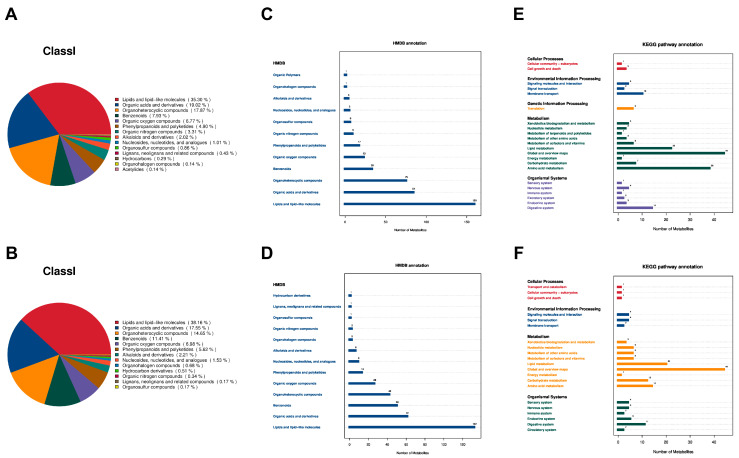
Identification and classification of all metabolites in plasma samples of Kazakh ewes. (**A**) Chemical classification of all detected metabolites under positive-ion mode and (**B**) under negative-ion mode. (**C**) HMDB (Human Metabolome Database) superclass annotations of all metabolites under positive-ion mode and (**D**) under negative-ion mode. In each panel (**A**–**D**), the left-hand side lists the classification categories (or annotation classes), and the right-hand side indicates the corresponding number of metabolites assigned to each category. (**E**) KEGG pathway enrichment annotations of all metabolites under positive-ion mode and (**F**) under negative-ion mode. For these two panels, the left side displays the enriched KEGG pathways and the right side shows the number of metabolites involved in each pathway.

Metabolomic analysis was performed on plasma samples collected from ewes following FSH or HMG treatment. In positive ion mode(Figure 2A), the detected metabolites comprised lipids and lipid-like molecules (35.30%), organic acids and derivatives (19.02%), organoheterocyclic compounds (17.87%), benzenoids (7.93%), organic oxygen compounds (6.77%), phenylpropanoids and polyketides (4.90%), organic nitrogen compounds (3.31%), alkaloids and derivatives (2.02%), nucleosides, nucleotides, and analogues (1.01%), organosulfur compounds (0.86%), lignans, neolignans, and related compounds (0.43%), hydrocarbons (0.29%), organohalogen compounds (0.14%), and acetylides (0.14%). In negative ion mode(Figure 2B), the metabolite composition included lipids and lipid-like molecules (38.16%), organic acids and derivatives (17.55%), organoheterocyclic compounds (14.65%), benzenoids (11.41%), organic oxygen compounds (6.98%), phenylpropanoids and polyketides (5.62%), alkaloids and derivatives (2.21%), nucleosides, nucleotides, and analogues (1.53%), organohalogen compounds (0.68%), hydrocarbon derivatives (0.51%), organic nitrogen compounds (0.34%), lignans, neolignans, and related compounds (0.17%), and organosulfur compounds (0.17%).

Annotation using the Human Metabolome Database (HMDB) and LIPIDMaps database revealed that the most abundant category in positive ion mode was(Figure 2C,E) “Lipids and lipid-like molecules” (159 metabolites), followed by “Organic acids and derivatives” (84 metabolites) and “Organoheterocyclic compounds” (75 metabolites). In negative ion mode(Figure 2D,F), “Lipids and lipid-like molecules” (132 metabolites) was also the most abundant, followed by “Organic acids and derivatives” (61 metabolites) and “Benzenoids” (50 metabolites). KEGG pathway annotation further categorized the identified metabolites into five major functional groups: Cellular Processes, Environmental Information Processing, Genetic Information Processing, Metabolism, and Organismal Systems. Specifically, metabolites in positive ion mode were primarily associated with cell growth and death, membrane transport, global and overview maps, amino acid metabolism, and the digestive system, whereas those in negative ion mode were mainly involved in signaling molecules and interaction, lipid metabolism, global and overview maps, and the digestive system.

### 3.3. Plasma Metabolomics Analysis

#### 3.3.1. Multivariate Statistical Analysis

**Figure 3:**
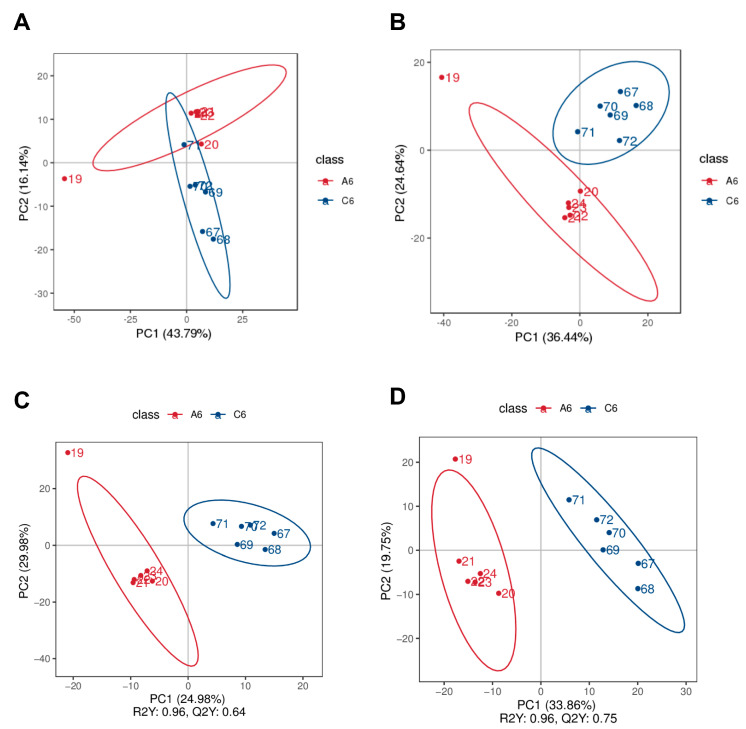
Multivariate statistical analysis results of differential metabolites in plasma samples of Kazakh ewes. Among them, the red represents the FSH group and the blue represents the HMG group. Principal component analysis (PCA) score plots of the experimental group and control group in positive ion mode (**A**) and negative ion mode (**B**). PLS-DA score plots of the experimental group and control group in positive-ion mode (**C**) and negative-ion mode (**D**).

Principal component analysis (PCA) was performed to evaluate the separation and clustering patterns between the FSH and HMG groups on day 6 post-insemination. In positive ion mode, PC1 and PC2 accounted for 43.79% and 16.14% of the total variance, respectively (Figure 3A). In negative ion mode, PC1 and PC2 explained 36.44% and 24.64% of the variance, respectively (Figure 3B). These results indicated that plasma metabolites on day 6 post-insemination exhibited significant separation between the two treatment groups, suggesting distinct metabolic profiles.

To further explore intergroup differences, partial least squares discriminant analysis (PLS-DA) was conducted. In positive ion mode, the PLS-DA model yielded R^2^Y = 0.96 and Q^2^ = 0.64 (Figure 3C), while in negative ion mode, the model yielded R^2^Y = 0.96 and Q^2^ = 0.75 (Figure 3D). The clear separation between groups in both ion modes confirmed that the PLS-DA models effectively distinguished the two treatment groups, demonstrating high reliability of the multivariate analysis.

#### 3.3.2. Analysis of Differentially Expressed Metabolites

**Figure 4:**
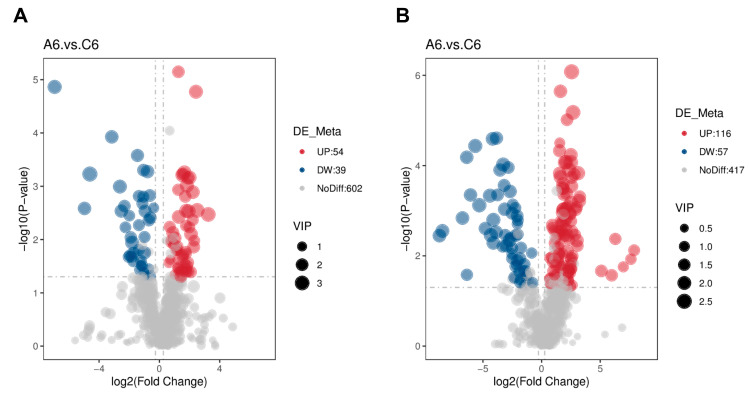
Analysis results of differential metabolites in plasma samples of Kazakh ewes. Volcano plots of differential metabolites in positive-ion mode (**A**) and negative-ion mode (**B**). The horizontal axis represents the fold change; points farther from the center indicate a larger fold change. The vertical axis represents the adjusted *p*-value; points closer to the top indicate more significant differences.

Based on standardized metabolite abundance data, the significance of intergroup differences was assessed using a two-tailed Student’s *t*-test to obtain *p*-values and calculate fold change (FC) values. These were combined with a PLS-DA model to derive variable importance scores (VIP). The criteria for identifying differentially expressed metabolites were as follows: VIP ≥ 1; *p* ≤ 0.05; and FC > 1.2 or <0.83. In samples collected on day 6 post-artificial insemination, a total of 1285 metabolites were identified in the FSH/HMG group: 93 differentially expressed metabolites were identified in positive ion mode, with 54 upregulated and 39 downregulated (Figure 4A); 173 differentially expressed metabolites were identified in negative ion mode, with 116 upregulated and 57 downregulated (Figure 4B).

Based on the FC values, the predominant differentially expressed metabolites in positive ion mode were Tiglylglycine, Tyrosyl-Leucine, 6-octenoylglycine, Ancistrolikokine A, etc. (Table 2); while the predominant differentially expressed metabolites in the negative ion mode were 11-Mercaptoundecanoic acid, 4-Dihydroboldenone, Terretrione B, 5b-Dihydrotestosterone, 3,4-Dimethyl-5-pentyl-2-furannonanoic acid, Alstonoxine B, and others (Table 3).

#### 3.3.3. Kegg Pathway Enrichment Analysis of Differentially Expressed Metabolites

**Figure 5:**
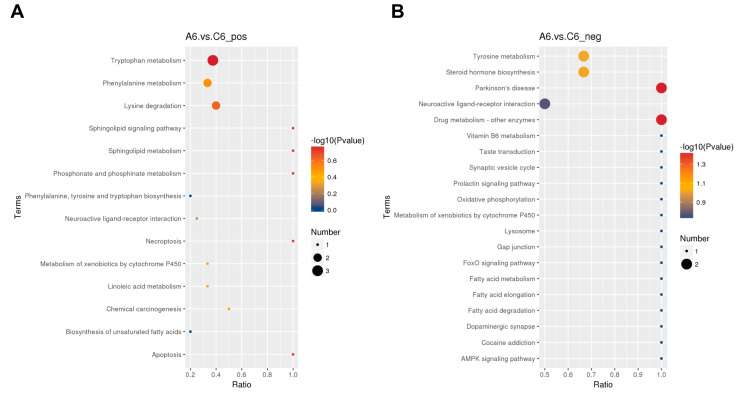
KEGG pathway analysis of differential metabolites in plasma samples of Kazakh ewes. (**A**) Top 14 KEGG enrichment pathways of differential metabolites in positive-ion mode. (**B**) Top 31 KEGG enrichment pathways of differential metabolites in negative-ion mode. In the figure, the x-axis represents pathway impact, the y-axis represents the *p*-value, and circles represent metabolic pathways. The color depth indicates the significance of metabolite changes in the pathway, and the circle size corresponds to the pathway score.

KEGG enrichment analysis was performed to identify the metabolic pathways associated with the differential metabolites. In positive ion mode, 14 significantly enriched pathways were identified, primarily including tryptophan metabolism, phosphonate and phosphinate metabolism, sphingolipid metabolism, sphingolipid signaling pathway, apoptosis, necroptosis, lysine degradation, and phenylalanine metabolism (Figure 5A). In negative ion mode, 31 significantly enriched pathways were detected, with the most prominent being drug metabolism—other enzymes, Parkinson’s disease, steroid hormone biosynthesis, tyrosine metabolism, neuroactive ligand-receptor interaction, fatty acid elongation, fatty acid degradation, and oxidative phosphorylation (Figure 5B). Among these, tyrosine metabolism, steroid hormone biosynthesis, Parkinson’s disease, neuroactive ligand-receptor interaction, and drug metabolism—other enzymes represented the most significantly enriched pathways.

#### 3.3.4. Cluster Analysis of Differentially Expressed Metabolites

**Figure 6:**
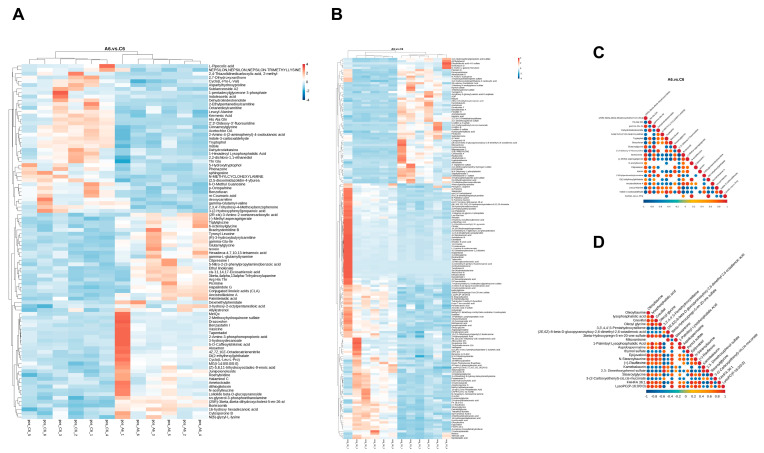
Differential analysis of plasma metabolites in Kazakh ewes after hormone treatment. (**A**) Heatmaps depicting the relative abundances of differentially accumulated metabolites under positive-ion and (**B**) negative-ion modes. In each heatmap, rows correspond to individual samples and columns to metabolites, both subjected to hierarchical clustering. Shorter branch lengths in the dendrograms indicate higher similarity among samples or metabolites. Horizontal comparisons across rows (samples) reveal the clustering trends of metabolite abundance among experimental groups. (**C**) Correlation matrices of the differentially accumulated metabolites under positive-ion and (**D**) negative-ion modes. Pearson correlation coefficients were computed for all pairwise metabolite combinations. The colour scale represents correlation strength: red indicates positive correlation approaching +1, blue indicates negative correlation approaching −1, and the intensity reflects the linear relationship. All correlations with a significance threshold of *p* < 0.05 are considered statistically significant. For clarity, the matrices display the top 20 metabolites with the smallest *p*-values (ranked in ascending order).

Hierarchical clustering analysis revealed distinct separation in the expression patterns of differential metabolites between the HMG and FSH groups in both positive and negative ion modes (Figure 6). Specifically, in negative ion mode, the HMG group exhibited significantly elevated ADP levels (*p* = 0.03), markedly downregulated hexadecanoic acid levels (*p* = 0.002), and significantly decreased 5-fluorouridine levels (Figure 6A,B). Pearson correlation analysis further revealed co-regulatory relationships among metabolites, with a strong positive correlation observed among glycerophospholipid metabolites, suggesting that these lipid molecules may participate in membrane remodeling and signal transduction through shared synthetic or degradative pathways.

#### 3.3.5. Analysis of Kegg Regulatory Networks for Differentially Expressed Metabolites

**Figure 7:**
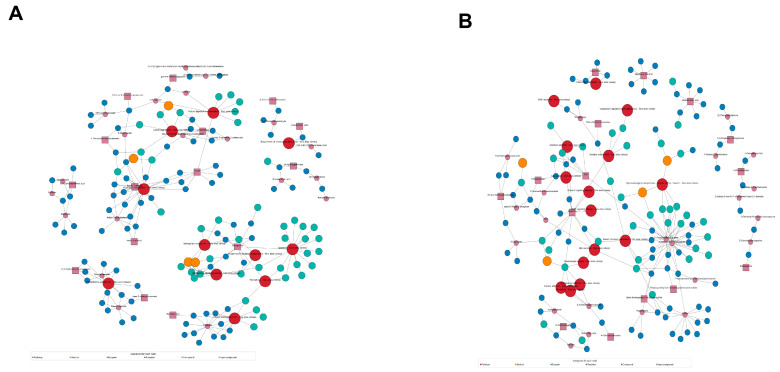
KEGG regulatory network of plasma metabolites in Kazakh sheep. (**A**) Positive-ion mode and (**B**) negative-ion mode. Red circles represent metabolic pathways; yellow circles represent molecular module information of a class of substances; green circles represent regulatory enzyme information related to a certain metabolite; purple circles represent background metabolites of a metabolic pathway; blue circles represent chemical interaction reactions of metabolites; purple squares represent differential metabolites identified in this comparison.

A KEGG-based regulatory network was constructed to visualize the interactions among differential metabolites and their associated biological pathways (Figure 7A,B). The network highlighted key metabolites—including ADP, hexadecanoic acid, and 5-fluorouridine—along with their corresponding pathway nodes and regulatory enzyme interactions, providing a comprehensive view of the metabolic alterations induced by HMG treatment.

### 3.4. Measurement of Plasma Reproductive Hormone Levels

**Figure 8:**
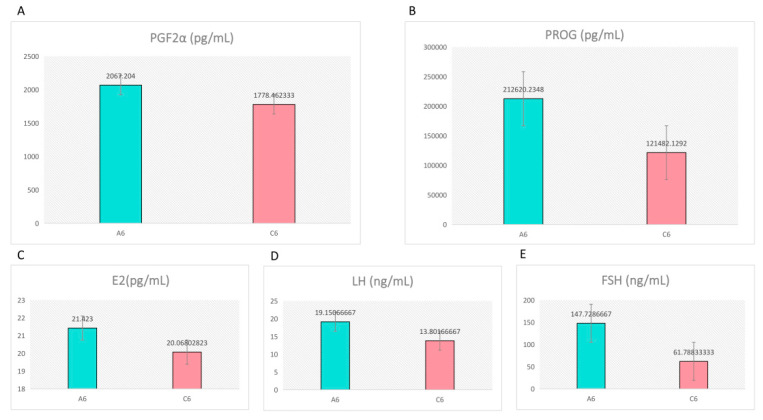
Comparison of reproductive hormone levels between FSH and HMG groups. All values are presented as mean ± standard deviation (SD). In panels (**A**–**C**), the concentration unit is pg/mL. In panels (**D**,**E**), the concentration unit is ng/mL.

As shown in Figure 8, compared with group FSH, there was a significant difference in plasma PROG levels in Group HMG Kazakh ewes on day 6 after artificial insemination (*p* = 0.01), with lower concentrations; however, there were no significant differences in the levels of PGF2α, E2, FSH, and LH (*p* > 0.05).

## 4. Discussion

Synchronized estrus and artificial insemination are now widely adopted in sheep breeding operations, and precise hormonal regulation during these procedures has become a standard component of reproductive management in the sheep industry [19]. Nevertheless, the effects of human menopausal gonadotropin (HMG) on embryo yield and quality remain a subject of debate, with reports across different studies showing considerable variability. In the present study, we compared the number of viable embryos recovered on Day 6 after artificial insemination between ewes subjected to HMG versus FSH superovulation protocols. Our results demonstrate that the HMG group produced significantly more viable embryos per donor (5.36 ± 3.29, *n* = 11) than the FSH group (2.91 ± 2.60, *n* = 35) (*p* < 0.05). Notably, only 12 non-viable embryos were recorded in the HMG group, whereas 129 non-viable embryos were observed in the FSH group (*p* < 0.05). Collectively, these findings indicate that HMG confers significant advantages in improving embryo quality, reducing embryonic degeneration, and increasing the yield of viable embryos following superovulation in sheep. Thus, HMG represents a viable and effective alternative to FSH for optimizing superovulation protocols in ovine embryo transfer programs.

To elucidate the molecular mechanisms by which HMG increases the number of viable embryos, we performed untargeted metabolomic profiling on plasma samples collected from both treatment groups. This analysis identified three key differential metabolites in the HMG group on day 6 post-insemination: 5-fluorouridine, adenosine diphosphate (ADP), and hexadecanoic acid, which were primarily enriched in pathways related to drug metabolism (other enzymes), Parkinson’s disease, tyrosine metabolism, and neuroactive ligand-receptor interactions. Concurrent hormone measurements revealed that only progesterone (PROG) levels differed significantly between the two groups, with no notable differences observed in FSH, LH, E2, or PGF2α concentrations.

Notably, although the HMG group produced significantly more viable embryos than the FSH group, this advantage did not translate into improved pregnancy rates or lambing rates. This apparent discrepancy suggests that HMG primarily optimizes the maternal endocrine and metabolic microenvironment during oocyte maturation and early embryonic development, rather than directly affecting post-implantation developmental processes. The precise mechanisms underlying this dissociation between embryo quality and reproductive outcomes warrant further investigation.

Plasma ADP levels were significantly elevated in the HMG group compared with the FSH group (*p* = 0.03). ADP is a purine nucleotide generated primarily through ATP dephosphorylation, a hydrolysis reaction that releases energy and positions ADP as a central regulator of intracellular energy homeostasis [20,21,22,23]. During early embryonic development, ADP and its dynamic metabolic turnover are indispensable for the energetically demanding transition from zygote to blastocyst, directly influencing embryo survival, developmental progression, and the ultimate yield of viable embryos [24]. Previous studies have established that ADP and ATP concentrations rise markedly to meet the bioenergetic demands of embryonic morphogenesis [25]. In cattle, for instance, promoting β-oxidation to drive ATP production—which consumes ADP as a substrate—synergistically enhances embryonic development to the morula stage [26]. Beyond its role in energy metabolism, ADP serves as the obligate substrate for poly(ADP-ribosyl)ation (PARP)-mediated DNA damage repair [27], a process of particular importance in early embryos, which exhibit inherently poor genomic stability and heightened sensitivity to DNA lesions [28,29]. PARP signaling is essential for normal DNA replication and cell division during early embryogenesis; its pharmacological inhibition leads to developmental arrest [30]. Mice with functional deficiencies in PARP-1 and Ku80 exhibit increased early embryonic mortality due to genomic instability and apoptosis [28], and doxorubicin-induced PARP inhibition similarly triggers apoptosis in blastocysts [31]. Collectively, these lines of evidence suggest that the HMG-induced elevation of ADP in the HMG group likely serves a dual function: it reinforces ATP regeneration to sustain embryonic energy homeostasis, while simultaneously potentiating DNA repair capacity to mitigate apoptosis. This dual mechanism provides a compelling molecular basis for the increased number of viable embryos observed following HMG treatment.

5-Fluorouridine, one of the key differential metabolites identified in this study, is a fluoropyrimidine nucleoside analog [32,33,34]. Upon intracellular accumulation, it disrupts ribosomal RNA maturation and protein synthesis following its incorporation into RNA, thereby exerting cytotoxic effects that impair embryonic development [35,36,37]. Due to its potent interference with RNA metabolism, 5-fluorouridine exhibits marked embryotoxicity and developmental toxicity [38]. In clinical, 5-fluorouracil (5-FU)—the precursor of 5-fluorouridine—is employed as an apoptosis inducer, and its administration has been shown to trigger apoptosis in specific embryonic regions at concentrations exceeding normal physiological thresholds [39,40,41]. The reproductive and embryotoxic effects of 5-fluorouridine and its precursors have been extensively documented. In toxicity studies, 5-FU concentrations as low as 0.01 µM have been reported to induce morphological abnormalities and genotoxicity [38]. In murine models, 5-fluorouridine inhibits oocyte maturation and preimplantation embryonic development, damages preantral follicles, and causes fetal malformations and prenatal death [42,43], with its toxic effects manifesting as delayed or arrested embryonic development [36]. Furthermore, fluoropyrimidine analogs have been shown to arrest embryonic development at the early gastrula stage in relevant embryotoxicity models [34]. Consistent with these findings, the elevated 5-fluorouridine levels observed in the FSH group likely contributed to the reduced number of viable embryos by inducing aberrant apoptosis and disrupting RNA metabolism and protein synthesis. In contrast, HMG treatment effectively downregulated this embryotoxic metabolite, thereby eliminating its deleterious interference and creating a more favorable molecular environment for oocyte maturation and early embryogenesis.

Palmitic acid (hexadecanoic acid), a saturated long-chain fatty acid, is known to exert lipotoxicity at elevated concentrations, thereby impairing oocyte maturation and early embryonic development [44,45]. In the present study, plasma palmitic acid levels were significantly downregulated in the HMG group compared with the FSH group (*p* = 0.002), suggesting that HMG treatment effectively alleviates lipotoxic stress during the peri-implantation period. At the cellular level, high concentrations of palmitic acid induce oxidative stress in oocytes and embryonic cells [44], disrupt mitochondrial membrane potential, and activate endoplasmic reticulum stress pathways [46,47]. These perturbations collectively reduce oocyte fertilization rates in vitro and compromise blastocyst development. In livestock species, including pigs and cattle, elevated palmitic acid levels have been shown to activate pro-inflammatory and pro-apoptotic signaling cascades, such as the TNF-α and NF-κB pathways, leading to diminished blastocyst formation rates [44,48]. Moreover, excessive palmitic acid exposure may induce aberrant changes in the embryonic transcriptome and DNA methylation patterns, further compromising developmental competence [45]. Furthermore, a significant negative correlation has been reported between palmitic acid (hexadecanoic acid) concentrations in human follicular fluid and the proportion of high-quality embryos, indicating that the lipotoxic effects of this metabolite are conserved across mammalian species [49]. In the present study, HMG treatment effectively attenuated lipotoxic damage to Kazakh sheep oocytes by reducing plasma palmitic acid concentrations, thereby preserving normal energy metabolism and physiological function in embryonic cells. Moreover, this reduction likely mitigated palmitic acid-induced apoptosis and inflammatory responses, creating a more favorable metabolic microenvironment for embryonic development. Collectively, these findings suggest that HMG-mediated downregulation of palmitic acid contributes to the amelioration of lipotoxic injury, thereby creating a more favorable metabolic milieu for oocyte maturation and early embryogenesis.

Progesterone, a steroid hormone primarily secreted by luteal cells in the ovaries [50], plays a critical role in regulating oocyte maturation [51]. In the present study, plasma progesterone concentrations in the HMG group were significantly lower than those in the FSH group (*p* = 0.01), suggesting that HMG treatment may mitigate the detrimental effects of premature progesterone elevation during the follicular phase on subsequent embryo quality.

Two lines of evidence support this interpretation. First, in human assisted reproductive technology, premature progesterone rise during the follicular phase (≥1.5 ng/mL) has been shown to significantly compromise oocyte and embryo quality, leading to reduced clinical pregnancy rates, embryo transfer success, and live birth rates, as well as delayed blastocyst development [52,53]. Moreover, an elevated progesterone-to-follicle ratio markedly diminishes embryonic chromosomal euploidy rates; even when comparable numbers of embryos are retrieved, the proportion of high-quality, viable embryos is substantially reduced [54,55,56]. Second, studies in bovine superovulation models have demonstrated that although the total embryo yield was slightly lower in the low-progesterone group, the proportions of Grade 1 quality embryos and late-stage transferable embryos were significantly increased, confirming that a moderately low progesterone environment is more conducive to the production of high-quality embryos [57].

In our study, progesterone levels in the FSH group were significantly elevated, likely reflecting premature luteinization during the follicular phase. This premature luteinization may have altered the follicular microenvironment, adversely affecting the final maturation of oocytes and the timing of ovulation, ultimately leading to reduced embryo quality and a decrease in the number of viable embryos. In contrast, HMG—which possesses both FSH and LH biological activities—may, through its LH component, finely regulate granulosa cell function and modulate the activity of key enzymes involved in progesterone synthesis, thereby maintaining progesterone concentrations within an optimal physiological range. Such regulation would prevent premature luteinization during the follicular phase, preserve the endocrine microenvironment required for oocyte maturation, and ultimately improve oocyte and embryo quality [58,59,60,61].

HMG significantly increased the number of usable embryos by upregulating ADP—a metabolite that plays a crucial role in embryonic energy metabolism and DNA repair—while concurrently downregulating the embryotoxic metabolites 5-fluorouridine and palmitic acid (hexadecanoic acid), and maintaining progesterone at an appropriate physiological level.

## 5. Conclusions

In conclusion, the HMG superovulation protocol significantly increased the number of usable embryos and reduced the number of non-usable embryos compared with the FSH protocol. Mechanistically, HMG appears to enhance embryo quality through coordinated regulation of progesterone, ADP, 5-fluorouridine, and palmitic acid, thereby improving the metabolic and endocrine microenvironment for embryonic development. These findings support HMG as an effective alternative to FSH for optimizing superovulation in sheep. Nevertheless, although the hMG group yielded a greater number of transferable embryos, this improved embryo collection outcome was not reflected in an elevated pregnancy rate.

## Figures and Tables

**Table 1:** Statistical data on embryo flushing following superovulation in sheep.

Group	HMG ^(C6)^	FSH ^(A6)^
Donor number	11	35
Total viable embryos	59	102
Average viable embryos	5.36 ± 3.29 *	2.91 ± 2.60
Total embryos	71	231
Average total embryos	6.45 ± 3.50	6.60 ± 4.29
Number of corpora lutea	125	302
Average number of corpora lutea	11.36 ± 6.12	8.62 ± 5.68
Number of non-viable embryos	12 *	129
Number of Ewes with Embryo Transfer	33	99
Number of Ewes Tested for Pregnancy	27	94
Number of Pregnant Ewes	17	54
Pregnancy Rate Detected by B-ultrasound	62.96%	57.45%
Number of Lambing Ewes	22	51
Lambing Rate	51.52%	66.67%

An asterisk (*) indicates *p* < 0.05. Distribution of statistical data for Kazakh ewes treated with HMG and FSH. Among these, the non-viable embryos included cleavage embryos (FSH group: 24; HMG group: 0), degenerated embryos (FSH group: 48; HMG group: 0), unfertilized oocytes (FSH group: 16; HMG group: 11), zonae pellucidae (FSH group: 25; HMG group: 1), and Grade C morulae (FSH group: 16; HMG group: 0).Superscript C6 represents the sixth day after artificial insemination in the HMG group, while superscript A6 denotes the sixth day after artificial insemination in the FSH group.

**Table 2:** Top 10 dominant differential metabolites in positive ion plasma of FSH/HMG.

Metabolite	Molecular Formula	Mean Plasma Abundance (FSH)	Mean Plasma Abundance (HMG)	FC	*p* Value	VIP
Tiglylglycine Tyrosyl-Leucine 6-octenoylglycine Ancistrolikokine A (R)-3-hydroxy butyrylcarnitine 3beta,4alpha,13alpha- Trihydroxylupanine N-acetylleucine 2-Methoxyhy droquinone sulfate Citpressine I (Z)-5,8,11- trihydroxyoctadec- 9-enoic acid	C7H11NO3 C15H22N2O4 C10H17NO3 C25H29NO4 C11H21NO5 C15H24N2O4 C8H15NO3 C7H8O6S C16H15NO5 C18H34O5	21,801,132.45 11,403,497.72 21,351,573.65 13,798,666.83 19,211,879.38 46,946,725.68 6,5850,297.21 2,830,404.751 5,391,999.422 4,764,189.451	2,305,230.18 2,002,171.489 4,413,762.955 2,960,500.972 4,483,435.397 11,131,659.39 16,399,758.09 712,601.1054 1,452,958.398 1,287,494.736	9.45 5.69 4.83 4.66 4.28 4.21 4.01 3.97 3.71 3.70	0.0033 0.0028 0.0138 0.0012 0.0079 0.0406 0.0006 0.0059 0.0006 0.0270	3.0 3.0 2.1 2.5 2.5 1.9 2.5 1.5 2.1 1.9

Statistics on the top ten cations in plasma from HMG- and FSH-treated Kazakh ewes.

**Table 3:** Top 10 dominant differential metabolites in negative ion plasma of FSH/HMG.

Metabolite	Molecular Formula	Mean Plasma Abundance (FSH)	Mean Plasma Abundance (HMG)	FC	*p* Value	VIP
11- Mercaptoun decanoic acid 4-Dihydro boldenone Terretrione B 5b-Dihydro testosterone 3,4-Dimethyl- 5-pentyl-2- furannonanoic acid Alstonoxine B N-Palmitoyl Lysine Isolysergol FAHFA 36:1 Etomidate	C11H22O2S C19H28O2 C17H22N2O3 C19H30O2 C20H34O3 C19H26N2O3 C22H44N2O3 C16H18N2O C36H68O4 C14H16N2O2	22,618,733.58 22,464,060.27 22,580,454.67 23,959,070.3 22,870,313.07 23,936,707.38 4,774,232.324 2,238,045.236 29,789,579.9 13,068,866.7	95,367.15196 114,558.7661 180,385.1207 306,494.8032 363,743.9901 700,316.5143 501,136.7994 244,551.2219 3,454,669.209 1,562,528.455	237 196 125 78.1 62.8 34.1 9.52 9.15 8.62 8.36	0.0075 0.0118 0.0175 0.0042 0.0269 0.0215 0.0004 0.0006 0.0001 0.0059	1.3 1.1 1.0 1.4 1.5 1.5 1.6 2.1 2.1 1.4

Statistics on the top ten anionic metabolites in plasma from Kazakh ewes treated with HMG and FSH.

## Data Availability

The data presented in this study are available on request from the corresponding author.

## References

[1] Betteridge K.J. (2003). A history of farm animal embryo transfer and some associated techniques. Anim. Reprod. Sci..

[2] Wu H.M., Chang H.M., Leung P.C.K. (2021). Gonadotropin-releasing hormone analogs: Mechanisms of action and clinical applications in female reproduction. Front. Neuroendocrinol..

[3] Garcia R., Filho R.A.A., Sitó-Silva L., Denadai R., Codognoto V., Salgado L., Brochine S., Oba E. (2024). Effect of pre-treatment with deslorelin on the ovarian response of ewes superovulated with FSH. Reprod. Domest. Anim..

[4] Haresign W. (1992). Manipulation of reproduction in sheep. J. Reprod. Fertil..

[5] Bartlewski P.M., Seaton P., Franco Oliveira M.E., Kridli R.T., Murawski M., Schwarz T. (2016). Intrinsic determinants and predictors of superovulatory yields in sheep: Circulating concentrations of reproductive hormones, ovarian status, and antral follicular blood flow. Theriogenology.

[6] Shi J.M., Yi J.Y., Tian X.Z., Wang F., Lian Z.X., Han H.B., Fu J.C., Lv W.F., Liu G.S. (2015). Effects of seasonal changes on the ovulation rate and embryo quality in superovulated Black Suffolk ewes. Neuro Endocrinol. Lett..

[7] Quan F., Zhang Z., An Z., Hua S., Zhao X., Zhang Y. (2011). Multiple factors affecting superovulation in Poll Dorset in China. Reprod. Domest. Anim..

[8] Luna-Palomera C., Macías-Cruz U., Sánchez-Dávila F. (2019). Superovulatory response and embryo quality in Katahdin ewes treated with FSH or FSH plus eCG during non-breeding season. Trop. Anim. Health Prod..

[9] Chumchai R., Ratsiri T., Ratchamak R., Vongpralub T., Boonkum W., Chankitisakul V. (2021). Ovarian responses and FSH profiles at superovulation with a single epidural administration of gonadotropin in the Thai-Holstein crossbreed. Anim. Reprod..

[10] Manjunatha B.M., Al-Hosni A., Al-Bulushi S. (2019). Simplified superovulation protocols in dromedary camels (*Camelus dromedarius*). Theriogenology.

[11] Forcada F., Ait Amer-Meziane M., Abecia J.A., Maurel M.C., Cebrián-Pérez J.A., Muiño-Blanco T., Asenjo B., Vázquez M.I., Casao A. (2011). Repeated superovulation using a simplified FSH/eCG treatment for in vivo embryo production in sheep. Theriogenology.

[12] Wang X.L., El-Gayar M., Knight P.G., Holtz W. (2009). The long-term effect of active immunization against inhibin in goats. Theriogenology.

[13] Yu L., Xia Q., Sun Z., Song J. (2022). Efficacy of Acupoint Application on In Vitro Fertilization Outcome in Patients with Polycystic Ovary Syndrome: A UHPLC-MS-Based Metabolomic Study. Evid.-Based Complement. Altern. Med. eCAM.

[14] Liu Y., Fan X., Pang H., Liu S., Wang B., Wang C., Yan Q., Song W., Hu Z., Liu Q. (2023). Metabolomics Identifies a Panel of Diagnostic Biomarkers for Early Human Embryonic Development Arrest. J. Proteome Res..

[15] Gibreel A., Bhattacharya S. (2010). Recombinant follitropin alfa/lutropin alfa in fertility treatment. Biol. Targets Ther..

[16] Romero J.J., Liebig B.E., Broeckling C.D., Prenni J.E., Hansen T.R. (2017). Pregnancy-induced changes in metabolome and proteome in ovine uterine flushings. Biol. Reprod..

[17] Hornbeck P.V. (2015). Enzyme-Linked Immunosorbent Assays. Curr. Protoc. Immunol..

[18] Young V.P., Mariano M.C., Faure L., Spencer J.V. (2021). Detection of Cytomegalovirus Interleukin 10 (cmvIL-10) by Enzyme-Linked Immunosorbent Assay (ELISA). Methods Mol. Biol..

[19] Türk G., Gür S., Sönmez M., Bozkurt T., Aksu E.H., Aksoy H. (2008). Effect of exogenous GnRH at the time of artificial insemination on reproductive performance of Awassi ewes synchronized with progestagen-PMSG-PGF2alpha combination. Reprod. Domest. Anim..

[20] Goto S., Chuman H., Majima E., Terada H. (2002). How does the mitochondrial ADP/ATP carrier distinguish transportable ATP and ADP from untransportable AMP and GTP?Dynamic modeling of the recognition/translocation process in the major substrate binding region. Biochim. Biophys. Acta.

[21] Miller S.G., Hafen P.S., Brault J.J. (2019). Increased Adenine Nucleotide Degradation in Skeletal Muscle Atrophy. Int. J. Mol. Sci..

[22] Pani A.K., Jiao Y., Sample K.J., Smeyne R.J. (2014). Neurochemical measurement of adenosine in discrete brain regions of five strains of inbred mice. PLoS ONE.

[23] Sohrabi-Mahboub M., Jahangiri S., Farrokhpour H. (2019). Molecular dynamics simulation of the hydration of adenosine phosphates. J. Mol. Liq..

[24] Scantland S., Tessaro I., Macabelli C.H., Macaulay A.D., Cagnone G., Fournier É., Luciano A.M., Robert C. (2014). The adenosine salvage pathway as an alternative to mitochondrial production of ATP in maturing mammalian oocytes. Biol. Reprod..

[25] Pincetich C.A., Viant M.R., Hinton D.E., Tjeerdema R.S. (2005). Metabolic changes in Japanese medaka (Oryzias latipes) during embryogenesis and hypoxia as determined by in vivo 31P NMR. Comp. Biochem. Physiol. Toxicol. Pharmacol. CBP.

[26] Sutton-McDowall M.L., Feil D., Robker R.L., Thompson J.G., Dunning K.R. (2012). Utilization of endogenous fatty acid stores for energy production in bovine preimplantation embryos. Theriogenology.

[27] Grenier L., Robaire B., Hales B.F. (2012). The activation of DNA damage detection and repair responses in cleavage-stage rat embryos by a damaged paternal genome. Toxicol. Sci..

[28] Henrie M.S., Kurimasa A., Burma S., Ménissier-de Murcia J., de Murcia G., Li G.C., Chen D.J. (2003). Lethality in PARP-1/Ku80 double mutant mice reveals physiological synergy during early embryogenesis. DNA Repair.

[29] Kim D.H., Shin S.T., Lee H.T. (2017). 55 The Role of Poly (ADP-Ribose) Polymerases in Porcine Cumulus–Oocyte Complex During In Vitro Maturation and Embryonic Development. Reprod. Fertil. Dev..

[30] Imschenetzky M., Montecino M., Puchi M. (1993). Temporally different poly(adenosine diphosphate-ribosylation) signals are required for DNA replication and cell division in early embryos of sea urchins. J. Cell. Biochem..

[31] Wang Q.L., Sun S.C., Han J., Kwak Y.C., Kim N.H., Cui X.S. (2012). Doxorubicin induces early embryo apoptosis by inhibiting poly(ADP ribose) polymerase. Vivo.

[32] Semail N.-F., Abdul Keyon A.S., Saad B., Noordin S.S., Nik Mohamed Kamal N.N.S., Mohamad Zain N.N., Azizi J., Kamaruzaman S., Yahaya N. (2020). Analytical method development and validation of anticancer agent, 5-fluorouracil, and its metabolites in biological matrices: An updated review. J. Liq. Chromatogr. Relat. Technol..

[33] Naren G., Guo J., Bai Q., Fan N., Nashun B. (2022). Reproductive and developmental toxicities of 5-fluorouracil in model organisms and humans. Expert Rev. Mol. Med..

[34] Karnofsky D.A., Basch R.S. (1960). Effects of 5-fluorodeoxyuridine and related halogenated pyrimidines on the sand-dollar embryo. J. Biophys. Biochem. Cytol..

[35] McDowell W., Weckbecker G., Schwarz R.T. (1986). Uridine sugar nucleotides modified in their nucleoside moieties and their effect on lipid-linked saccharide formation in vitro. Biosci. Rep..

[36] Naren G., Li D., Xing D., Liu Y., Wang L., Fan N., Li H., Bai X., Zeng X., Wang J. (2025). Smug1 alleviates the reproductive toxicity of 5-FU through functioning in rRNA quality control. Sci. Rep..

[37] Nakagawa H., Yamada M., Fukushima M., Shimizu K., Ikenaka K. (1998). In vitro study on intrathecal application of 5-fluoro-2′-deoxyuridine (FdUrd) for meningeal dissemination of malignant tumor. NO Shinkei Geka. Neurol. Surg..

[38] Kovács R., Csenki Z., Bakos K., Urbányi B., Horváth Á., Garaj-Vrhovac V., Gajski G., Gerić M., Negreira N., López de Alda M. (2015). Assessment of toxicity and genotoxicity of low doses of 5-fluorouracil in zebrafish (*Danio rerio*) two-generation study. Water Res..

[39] Yamaguchi Y., Fukunaga Y., Takagi M., Saito T., Tamura K., Hoshiya T. (2021). Time-course changes in 5-fluorouracil-induced neural progenitor cell damages in the developing rat brain. J. Toxicol. Pathol..

[40] Yamaguchi Y., Aoki A., Fukunaga Y., Matsushima K., Ebata T., Ikeya M., Tamura K. (2009). 5-fluorouracil-induced histopathological changes in the central nervous system of rat fetuses. Histol. Histopathol..

[41] Kumar S., Aninat C., Michaux G., Morel F. (2010). Anticancer drug 5-fluorouracil induces reproductive and developmental defects in Caenorhabditis elegans. Reprod. Toxicol..

[42] Naren G., Wang L., Zhang X., Cheng L., Yang S., Yang J., Guo J., Nashun B. (2021). The reversible reproductive toxicity of 5-fluorouracil in mice. Reprod. Toxicol..

[43] Almeida J.Z., Lima L.F., Vieira L.A., Maside C., Ferreira A.C.A., Araújo V.R., Duarte A.B.G., Raposo R.S., Báo S.N., Campello C.C. (2021). 5-Fluorouracil disrupts ovarian preantral follicles in young C57BL6J mice. Cancer Chemother. Pharmacol..

[44] De Bie J., Smits A., Marei W.F.A., Leroy J.L.M.R. (2021). Capacity of Trolox to improve the development and quality of metabolically compromised bovine oocytes and embryos invitro during different windows of development. Reprod. Fertil. Dev..

[45] Marei W.F.A., Van Raemdonck G., Baggerman G., Bols P.E.J., Leroy J.L.M.R. (2019). Proteomic changes in oocytes after in vitro maturation in lipotoxic conditions are different from those in cumulus cells. Sci. Rep..

[46] Wang Y., Pope I., Brennan-Craddock H., Poole E., Langbein W., Borri P., Swann K. (2021). A primary effect of palmitic acid on mouse oocytes is the disruption of the structure of the endoplasmic reticulum. Reproduction.

[47] Van Hoeck V., Leroy J.L., Arias Alvarez M., Rizos D., Gutierrez-Adan A., Schnorbusch K., Bols P.E., Leese H.J., Sturmey R.G. (2013). Oocyte developmental failure in response to elevated nonesterified fatty acid concentrations: Mechanistic insights. Reproduction.

[48] Shi M., Sirard M.A. (2021). Transcriptome and epigenome analysis of porcine embryos from non-esterified fatty acid-exposed oocytes. Domest. Anim. Endocrinol..

[49] Mirabi P., Chaichi M.J., Esmaeilzadeh S., Ali Jorsaraei S.G., Bijani A., Ehsani M., Hashemi Karooee S.F. (2017). The role of fatty acids on ICSI outcomes: A prospective cohort study. Lipids Health Dis..

[50] Patel D.H., Fine L.M., Bernstein J.A. (2023). A focused report on progestogen hypersensitivity. Expert Rev. Clin. Immunol..

[51] Keyser S., van der Horst G., Maree L. (2021). Progesterone, Myo-Inositol, Dopamine and Prolactin Present in Follicular Fluid Have Differential Effects on Sperm Motility Subpopulations. Life.

[52] George J.S., Keefe K.W., Lanes A., Yanushpolsky E. (2023). Premature progesterone elevation during the early and mid-follicular phases in fresh in vitro fertilization (IVF) cycles is associated with lower live birth, clinical pregnancy, and implantation rates. J. Assist. Reprod. Genet..

[53] Li J., Cui Y., Shi H., Bu Z., Wang F., Sun B., Zhang Y. (2023). Effects of trigger-day progesterone in the preimplantation genetic testing cycle on the embryo quality and pregnancy outcomes of the subsequent first frozen-thawed blastocyst transfer. Front. Endocrinol..

[54] Ali K.I.A., Lawrenz B., Shanker U., Ruiz F., El-Damen A., ElKhatib I., Fatemi H., De Munck N. (2023). The Ratio of Serum Progesterone (P4) to the Number of Follicles (P4/follicle) is a More Objective Parameter for Euploidy Rate as Compared to Systemic Progesterone Levels. Reprod. Sci..

[55] Woo J., Kwon H., Choi D., Park C., Kim J., Shin J., Kim J., Kang Y.J., Koo H. (2022). Effects of Elevated Progesterone Levels on the Day of hCG on the Quality of Oocyte and Embryo. J. Clin. Med..

[56] Villanacci R., Buzzaccarini G., Marzanati D., Vanni V.S., De Santis L., Alteri A., Candiani M., Pagliardini L., Papaleo E. (2023). Delayed blastocyst development is influenced by the level of progesterone on the day of trigger. J. Assist. Reprod. Genet..

[57] Wiley C., Jahnke M., Redifer C., Gunn P.J., Dohlman T. (2019). Effects of endogenous progesterone during ovarian follicle superstimulation on embryo quality and quantity in beef cows. Theriogenology.

[58] Pan Y., Pan C., Zhang C. (2024). Unraveling the complexity of follicular fluid: Insights into its composition, function, and clinical implications. J. Ovarian Res..

[59] McLachlan R.I., Cohen N.L., Vale W.W., Rivier J.E., Burger H.G., Bremner W.J., Soules M.R. (1989). The importance of luteinizing hormone in the control of inhibin and progesterone secretion by the human corpus luteum. J. Clin. Endocrinol. Metab..

[60] Tatone C., Benedetti E., Vitti M., Di Emidio G., Ciriminna R., Vento M.E., Cela V., Borzì P., Carta G., Lispi M. (2016). Modulating Intrafollicular Hormonal Milieu in Controlled Ovarian Stimulation: Insights From PPAR Expression in Human Granulosa Cells. J. Cell. Physiol..

[61] Beckers N.G., Macklon N.S., Eijkemans M.J., Ludwig M., Felberbaum R.E., Diedrich K., Bustion S., Loumaye E., Fauser B.C. (2003). Nonsupplemented luteal phase characteristics after the administration of recombinant human chorionic gonadotropin, recombinant luteinizing hormone, or gonadotropin-releasing hormone (GnRH) agonist to induce final oocyte maturation in in vitro fertilization patients after ovarian stimulation with recombinant follicle-stimulating hormone and GnRH antagonist cotreatment. J. Clin. Endocrinol. Metab..

